# High-speed atomic force microscopy reveals a surface-catalyzed elongation mechanism of the fungal functional amyloid hydrophobin RolA

**DOI:** 10.1073/pnas.2523502123

**Published:** 2026-02-12

**Authors:** Nao Takahashi, Tatsuya Kimura, Yuki Terauchi, Takumi Tanaka, Natsuki Abe, Akira Yoshimi, Takahiro Watanabe-Nakayama, Keietsu Abe

**Affiliations:** ^a^Major of Agricultural Chemistry, Graduate School of Agricultural Science, Tohoku University, Sendai, Miyagi 980-8572, Japan; ^b^World Premier International Research Center Initiative Nano Life Science Institute, Kanazawa University, Kanazawa, Ishikawa 920-1192, Japan; ^c^Research Center for Thermotolerant Microbial Resources, Yamaguchi University, Yamaguchi, Yamaguchi 753-8511, Japan; ^d^Division of Environmental Science and Technology, Graduate School of Agriculture, Kyoto University, Kyoto 606-8502, Japan; ^e^Department of Natural Resources, Graduate School of Global Environmental Studies, Kyoto University, Kyoto 606-8501, Japan

**Keywords:** functional amyloid, high-speed AFM, surface-catalyzed elongation, hydrophobin, filamentous fungi

## Abstract

Hydrophobins are functional amyloids conserved in filamentous fungi. They act as a protective coat in the fibrous form, called rodlet. Rodlets further assemble to form dense films where they are bundled and densely aligned, contributing to the hydrophobicity of the mycelium surface. The mechanism of this dense film formation is completely unknown. Here, we used high-speed atomic force microscopy to directly observe the structural dynamics of rodlet bundling and subsequent film formation by hydrophobin RolA from the industrial fungus **Aspergillus* oryzae* at a single-fibril level, and we revealed the film-formation mechanism. Rodlet elongation occurred at both ends and was discontinuous, alternating between periods when rodlets could elongate (growth state) and could not elongate (pause state). This suggests an equilibrium of two distinct structural states at the rodlet ends. We also identified a pathway, termed “surface-catalyzed elongation,” in which elongation is promoted by lateral interactions between bundled rodlets. Surface-catalyzed elongation decreased the energy barrier of both structural switching between growth and pause states and elongation at rodlet ends, doubling the elongation rate in bundled rodlets. The rodlet surface could be considered as a catalyst for the elongation of neighboring rodlets. Surface-catalyzed elongation could contribute to rodlet bundling, whereby rodlets tend to form oriented domain structures; our Monte Carlo simulations confirmed this. The concept we propose here provides a clear explanation of the mechanism by which rodlets form a dense coat on the cell surface.

## Significance

The fungal functional amyloid hydrophobin RolA self-assembles into a fibrous form called a rodlet, which functions as a protective coat, but the mechanism of rodlet formation remains largely unknown. Here, we used high-speed atomic force microscopy to observe rodlet formation at the single-fibril level in real time. We identified a phenomenon termed surface-catalyzed elongation, by which the bundling of rodlets promotes their elongation. This phenomenon increases the likelihood of rodlet alignment, resulting in the formation of a dense film similar to that observed on the actual cell surface. Our findings revealed rodlet formation kinetics driven by surface-catalyzed elongation and provide an important conceptual advance for understanding the rodlet film-formation mechanism.

Amyloid proteins assembled into cross-β fibrils, which can bundle to form larger aggregates (1, 2), are used beneficially as functional amyloids by bacteria, fungi, insects, and mammals to maintain physiological processes (3, 4), although amyloid fibrils are often associated with protein misfolding and neurodegenerative diseases. Hydrophobins are functional amyloids produced by filamentous fungi; they form amyloid-like fibrils called rodlets, which become ordered to form dense films and support fungal physiology (5, 6). Hydrophobins are low-molecular-weight amphiphilic proteins that adhere to the cell wall surface to coat it and make it hydrophobic. This surface modification helps to promote the air dispersibility of conidia and helps hyphae to adhere to hydrophobic surfaces such as plant leaves coated with wax esters (7, 8). Hydrophobins also have immunosilencing properties, because hydrophobin coating of the hyphae of pathogenic filamentous fungi prevents recognition by the host immune system (5, 9–11). Some hydrophobins may be involved in the degradation of solid polymers (12–15).

Hydrophobins are classified into several classes on the basis of their amino acid sequences and surface hydrophobicity. Class I hydrophobins self-assemble into rodlets. Hydrophobins typically contain three hydrophobic loops that form cross β-sheet structures, which assemble into bundled ordered rodlets on the conidial surface (5, 6, 16). In our previous studies, purified class I hydrophobin RolA from the industrial fungus **Aspergillus* oryzae* formed dense rodlet films at the air–water interface; the films were transferred to the substrate, substantially altering substrate wettability (17, 18). Therefore, rodlets are key factors that affect solid surface properties and may regulate biological functions of the fungal cell wall, although the mechanism of their formation remains largely unknown. Understanding the dynamics of rodlet film formation, whereby hydrophobins transition from monomers to rodlets, which then densely assemble into aligned films, is key to uncovering the diverse functions of hydrophobins.

Rodlet formation is thought to resemble usual amyloid fibril formation: it is initiated by nucleation, followed by autocatalytic growth as monomers bind to the rodlet ends, using the preformed rodlets as templates (14, 19). Previous studies that used thioflavin T (ThT) assays and atomic force microscopy (AFM) with coarse temporal resolution have provided macroscopic insights into rodlet film formation but have failed to reveal the detailed mechanism (5, 14, 16, 18). A comprehensive understanding of rodlet film formation requires elucidating not only the formation of individual rodlets but also their interactions and assembly. This necessitates high spatial and temporal resolution of real-time observations at the single-fibril level and quantitative analysis of local interactions.

In this study, we used high-speed (HS) AFM (20) to observe formation of the rodlets of hydrophobin RolA from *A. oryzae* and to directly measure the kinetics of rodlet formation at a single-fibril level. HS-AFM showed that they have a remarkable ability to elongate rapidly and discontinuously from both ends. The kinetics of elongation depended on the presence of neighboring rodlets, with elongation accelerating in their vicinity. The lateral interactions of rodlets led to their bundling, and our Monte Carlo simulations showed that this local reaction, detected by HS-AFM, contributed to the collective ordering of the dense rodlet film. These findings provide mechanistic insights into the principles governing rodlet film formation.

## Results

### Rodlet Formation in Bulk Solution.

To investigate structural changes in RolA associated with rodlet formation, we used circular dichroism (CD) spectroscopy. The CD spectrum of monomeric RolA had a minimum at approximately 205 nm (*SI Appendix*, Fig. S1*A*), consistent with our previous findings (13). Upon rodlet formation, the minimum shifted to approximately 220 nm (*SI Appendix*, Fig. S1*B*), indicating an increase in β-sheet content compared with that in the monomeric state (21). These results support the hypothesis that RolA undergoes rodlet formation through the establishment of intermolecular β-sheet structures.

To further elucidate the mechanism of rodlet formation, we performed a ThT fluorescence assay. The reaction curves did not have the characteristic sigmoidal shape typically observed in amyloid protein aggregation (19). Instead, an immediate increase in fluorescence intensity was observed without a lag phase, followed by a constant rate of fluorescence increase until saturation was reached (*SI Appendix*, Fig. S1*C*). Data fitting with Eq. **1** (*Materials and Methods*) provided good fits (*SI Appendix*, Fig. S1*D*). Model fitting showed that rodlet formation was well described by the dock–lock model and the absence of a rodlet-replication system, such as secondary nucleation (22, 23), as reported previously for other class I hydrophobin, MPG1 of *Magnaporthe oryzae* (14). Because the air–water interface plays a crucial role in the self-assembly of hydrophobins (14, 17, 18), we examined its effects on RolA self-assembly. No increase in ThT fluorescence was observed in the absence of an air–water interface. However, immediately after the introduction of an air–water interface, ThT fluorescence markedly increased (*SI Appendix*, Fig. S1*E*), indicating that the interface strongly promoted rodlet formation. In a seeding assay, the addition of 10% (v/v) seed caused an increase in ThT fluorescence in the absence of an air–water interface (*SI Appendix*, Fig. S1*F*). These results indicate that, although RolA does not form aggregation-competent species without an air–water interface, seeding can occur. However, this analysis did not provide further mechanistic insight into rodlet formation, highlighting the limitations of bulk solution measurements in elucidating the details of this process.

### HS-AFM Analysis of Rodlet Elongation.

To elucidate the details of the mechanism of rodlet elongation, we examined it at the single-fibril level by using HS-AFM. A small amount of rodlets was first immobilized on a hydrophobic silicon substrate, and then the substrate was immersed in a RolA monomer solution (7.35 µM) during the measurement. We chose a hydrophobic substrate for this analysis because RolA adsorbs to solid surfaces via hydrophobic interactions (24) and can form rodlets at hydrophobic–hydrophilic interfaces (17).

**Fig. 1.**
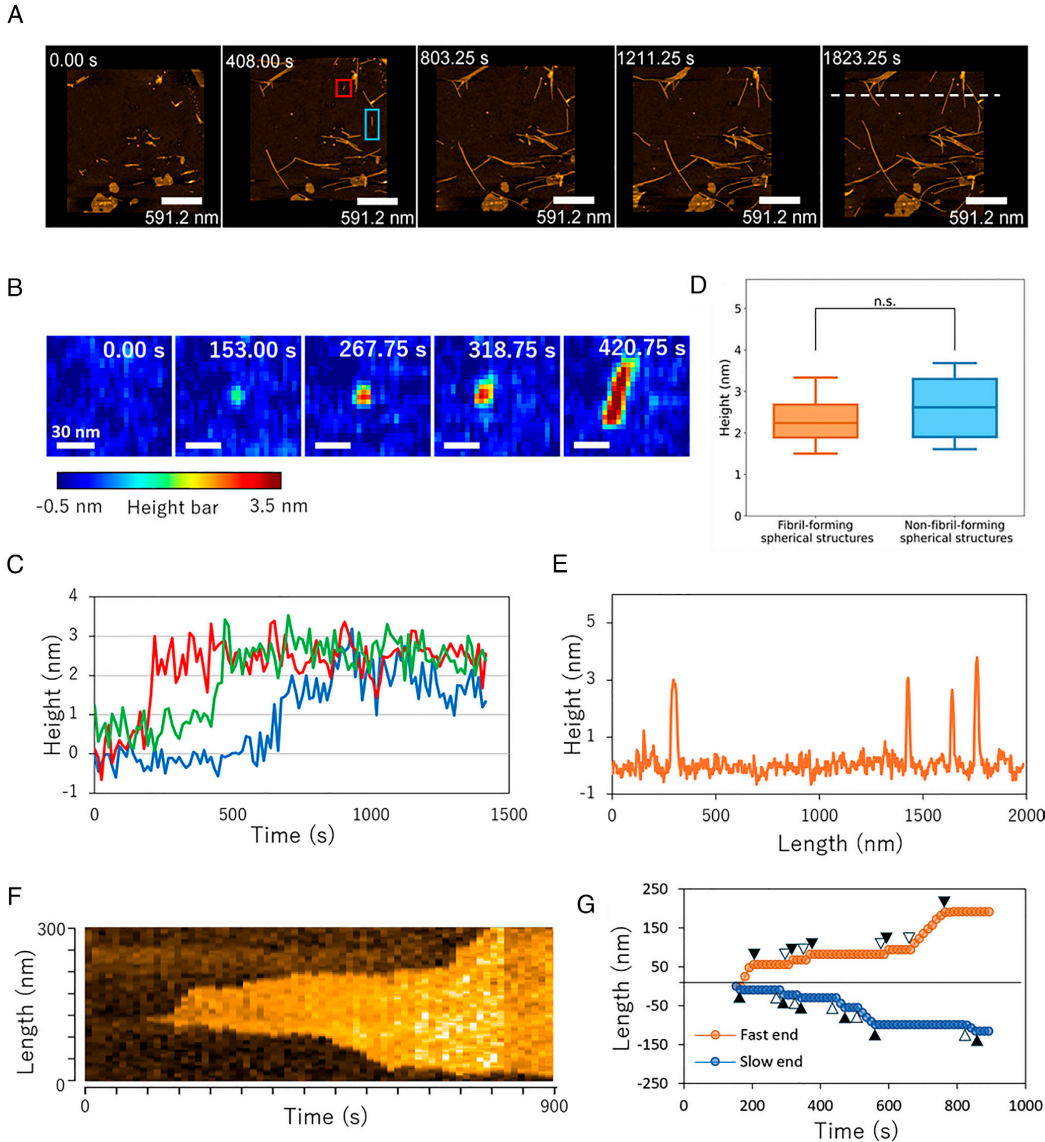
HS-AFM imaging of RolA rodlet formation. (*A*) Images taken during rodlet formation (scan size, 2 μm × 2 μm; pixels along the *X-*axis, 500; pixels along the *Y-*axis, 250). (*B*) Representative images of the area within the red square in (*A*), showing that a spherical structure was formed first, followed by rodlet formation. (*C*) Time evolution of the height of spherical structures, showing that they developed gradually before changing into rodlets. Three typical examples are shown. (*D*) Height of spherical structures. For the fibril-forming ones, the experimental data were fitted to a sigmoidal function, and the plateau value was used as the height (N = 17). For the non-fibril-forming ones, the height remained constant, and the average height from 1600s to 1800s was used (N = 12). Boxes extend from the 25th to 75th percentiles. The line in each box indicates the median. Whiskers reach out to the most distant point that is still within 1.5 times the interquartile range. Statistical significance was evaluated by using the Brunner–Munzel test. (*E*) Cross-section at the white dashed line in (*A*). (*F*) Representative kymograph of the area within the blue rectangle in (*A*), showing that the rodlet elongated from both ends in a stepwise manner. (*G*) Time evolution of the positions of rodlet ends, showing that both ends repeatedly grew and stopped. Black triangles, start points of dwell time; white triangles, start points of step time.

Measurements revealed frequent elongation of preexisting rodlets, as well as the formation and subsequent elongation of newly generated rodlets (de novo rodlets) (Fig. 1*A* and Movie S1). Spherical aggregates formed before rodlet emergence and served as rodlet precursors: their height gradually increased and reached 2.3 ± 0.1 nm (mean ± SE), followed by a structural transformation into rod-like formations, which subsequently elongated (Fig. 1 *B*–*D*). Spherical structures that were not converted into rodlets were also observed, with a height of 3.1 ± 0.4 nm (Fig. 1*D*). Cross-sectional analysis revealed that rodlets had an approximate height of 3 nm (Fig. 1*E*).

Preexisting rodlets initially formed at the air–water interface, whereas de novo rodlets appeared on the substrate. Because amyloid fibril elongation can vary depending on the structural properties of the template surface (25), we compared the elongation behavior of preexisting and de novo rodlets. HS-AFM video analysis revealed that rodlets alternated between periods of elongation and periods of length stabilization (Fig. 1 *F* and *G*). The apparent elongation rate, determined from the slope of a linear fit connecting the onset and termination points of rodlet elongation, was 7.9 ± 0.9 nm/min (mean ± SE) for preexisting rodlets and 11.6 ± 2.4 nm/min for de novo rodlets, with no statistically significant difference (*SI Appendix*, Fig. S2*A* and Table S1).

Dwell time (duration of no length change), step time (duration of elongation), and step size (elongation length per step time) for both preexisting and de novo rodlets followed a typical exponential distribution described by Eq. **2** (*Materials and Methods* and *SI Appendix*, Fig. S2 *B*–*D*). The mean dwell time was 61.0 s for preexisting rodlets vs. 75.6 s for de novo rodlets; step time was 29.7 s vs. 27.3 s; and step size was 26.5 nm vs. 19.2 nm. The step rate (elongation rate within a single elongation phase, calculated as step size/step time) was 9.0 ± 0.3 nm/min for preexisting rodlets and 8.8 ± 0.5 nm/min for de novo rodlets. No statistically significant differences were observed for any of these parameters (*SI Appendix*, Table S1). These results indicated that the elongation behavior of preexisting and de novo rodlets did not differ significantly.

### Rodlet Elongation from Both Ends.

**Fig. 2.**
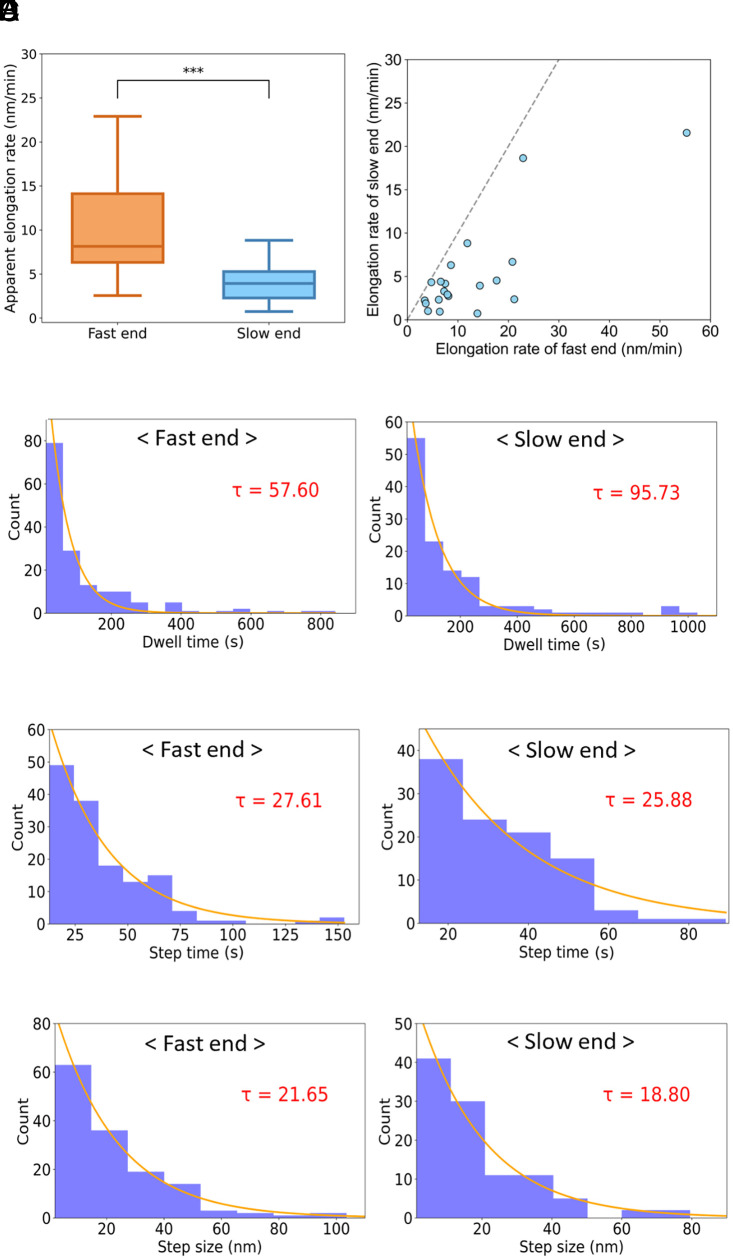
Elongation kinetics of fast and slow ends of rodlets. (*A*) Apparent elongation rate. Boxes extend from the 25th to 75th percentiles. The line in each box indicates the median. Whiskers reach out to the most distant point that is still within 1.5 times the interquartile range. Statistical significance was evaluated by using the Brunner–Munzel test (***P < 0.001). (*B*) Relationship between elongation rates of the fast and slow ends of the same rodlet. The dashed line is the line of equality. (*C*–*E*) Distributions of dwell time (*C*) and step time (*D*), and single-step size (*E*), with exponential fits (lines) giving the mean values of τ shown in each panel and *SI Appendix*, Table S2.

RolA rodlets actively elongated from both ends (Movie S2). First, we defined the fast and slow ends according to the apparent elongation rate. Some rodlets in dense regions already had one end in contact with a neighboring rodlet, preventing further elongation; such rodlets were excluded from the analysis. The apparent elongation rate was 12.6 ± 2.6 nm/min for fast ends and 5.2 ± 1.2 nm/min for slow ends; the difference was statistically significant (Fig. 2*A* and *SI Appendix*, Table S2). Because elongation rates varied considerably among individual rodlets (Fig. 2*B*), simply averaging the values at the fast and slow ends could have overestimated the difference between them. Therefore, we used a linear mixed effects model (26) for statistical analysis, treating the difference between the two ends as a fixed effect and the variability among individual rodlets as a random effect. We confirmed a statistically significant difference (*P* < 0.01) in the apparent elongation rate between the fast and slow ends. Even after accounting for variability among rodlets, this difference persisted, suggesting that it was not an artifact introduced during the analysis, but rather resulted from intrinsic differences between the two ends.

Although rodlet elongation was polar, the mean relative difference in apparent elongation rate [(Fast – Slow)/Fast] was only about 58%. Dwell time, step time, and step size for both fast and slow ends followed the typical exponential distribution described by Eq. **2** (Fig. 2 *C*–*E*). The mean dwell time was 57.6 s for fast ends vs. 95.7 s for slow ends; step time was 27.6 s vs. 25,9 s; step size was 21.7 nm vs. 18.8 nm; and mean step rate was 9.2 ± 0.4 nm/min vs. 8.5 ± 0.5 nm/min (*SI Appendix*, Table S2). Although no statistically significant differences were found for these four parameters (*SI Appendix*, Table S2), the dwell time tended to be shorter at the fast ends than at the slow ends.

### Promotion of Elongation in Bundled Rodlets.

**Fig. 3.**
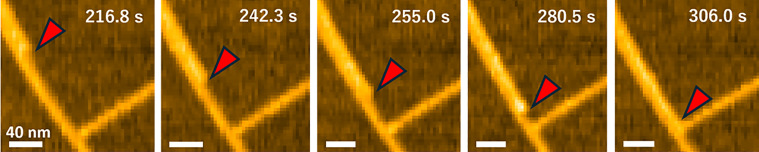
HS-AFM imaging of rodlet bundling. Representative images of a rodlet elongating along another rodlet. Red arrowheads indicate the tip of the rodlet during elongation.

**Fig. 4.**
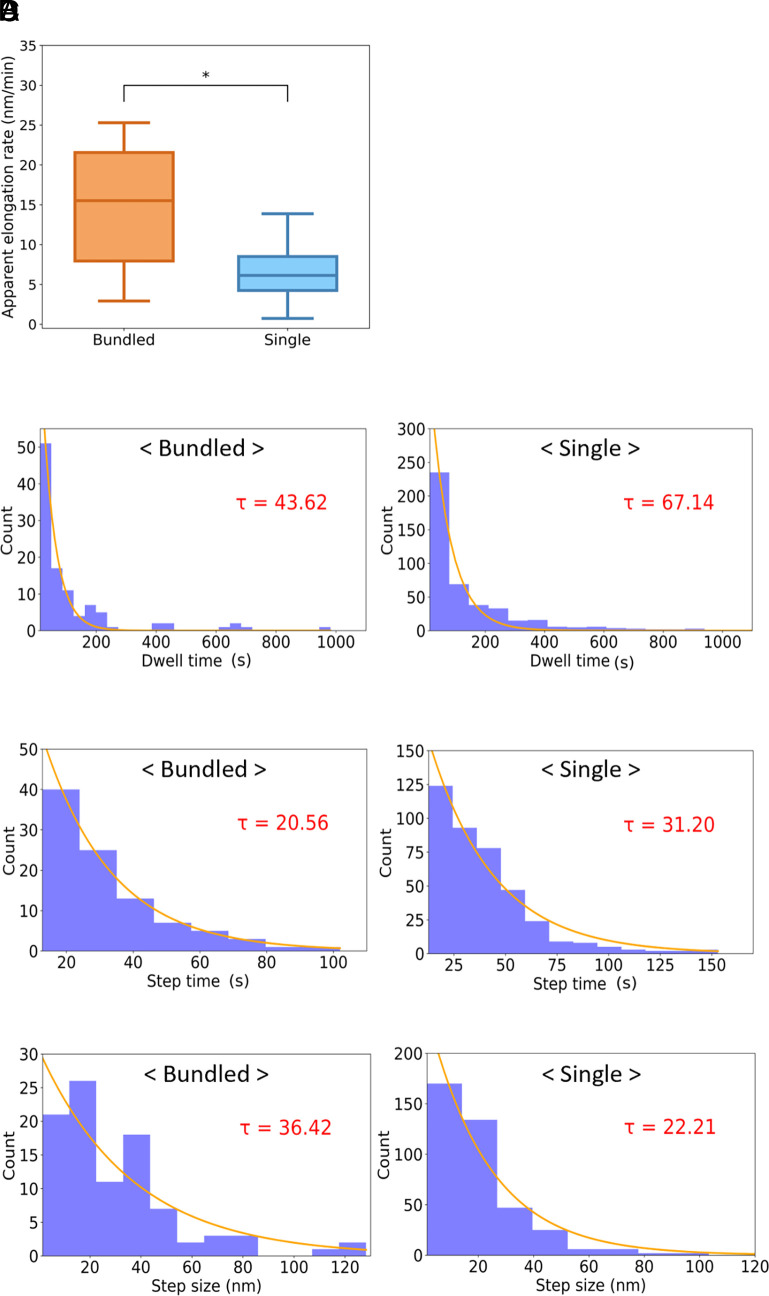
Elongation kinetics of bundled and single rodlets. (*A*) Apparent elongation rate. Boxes extend from the 25th to 75th percentiles. The line in each box indicates the median. Whiskers reach out to the most distant point that is still within 1.5 times the interquartile range. Statistical significance was evaluated by using the Brunner–Munzel test (*P < 0.05). (*B*–*D*) Distributions of dwell time (*B*), step time (*C*), and single-step size (*D*), with exponential fits (lines) giving the mean values of τ shown in each panel and *SI Appendix*, Table S3.

Multiple rodlets interacted laterally to form bundles, and individual rodlets elongated in coordination with others within the bundle (Fig. 3 and Movie S3). No dissociation of the bundles into individual rodlets was observed. Because lateral interactions between rodlets may influence their rate of elongation, we measured the elongation rates of both bundled and single rodlets; all elongating ends were included in the kinetic evaluation. The apparent elongation rate was significantly higher in bundled rodlets (13.9 ± 2.2 nm/min) than in single rodlets (7.4 ± 0.8 nm/min) (Fig. 4*A* and *SI Appendix*, Table S3).

In bundled rodlets, elongation and pause phases alternated and all data followed a characteristic exponential distribution described by Eq. **2** (Fig. 4 *B*–*D*). The mean dwell time was 43.6 s for bundled rodlets vs. 67.1 s for single rodlets; step time was 20.6 s vs. 31.2 s; step size was 36.4 nm vs. 22.2 nm; and mean step rate was 17.9 ± 2.0 nm/min vs. 10.4 ± 0.6 nm/min (Fig. 4 *B*–*D* and *SI Appendix*, Table S3); all these differences were statistically significant. These results suggest that the termini of bundled rodlets incorporate monomers significantly more efficiently than those of single rodlets.

### Simulations of Domain Formation.

**Fig. 5.**
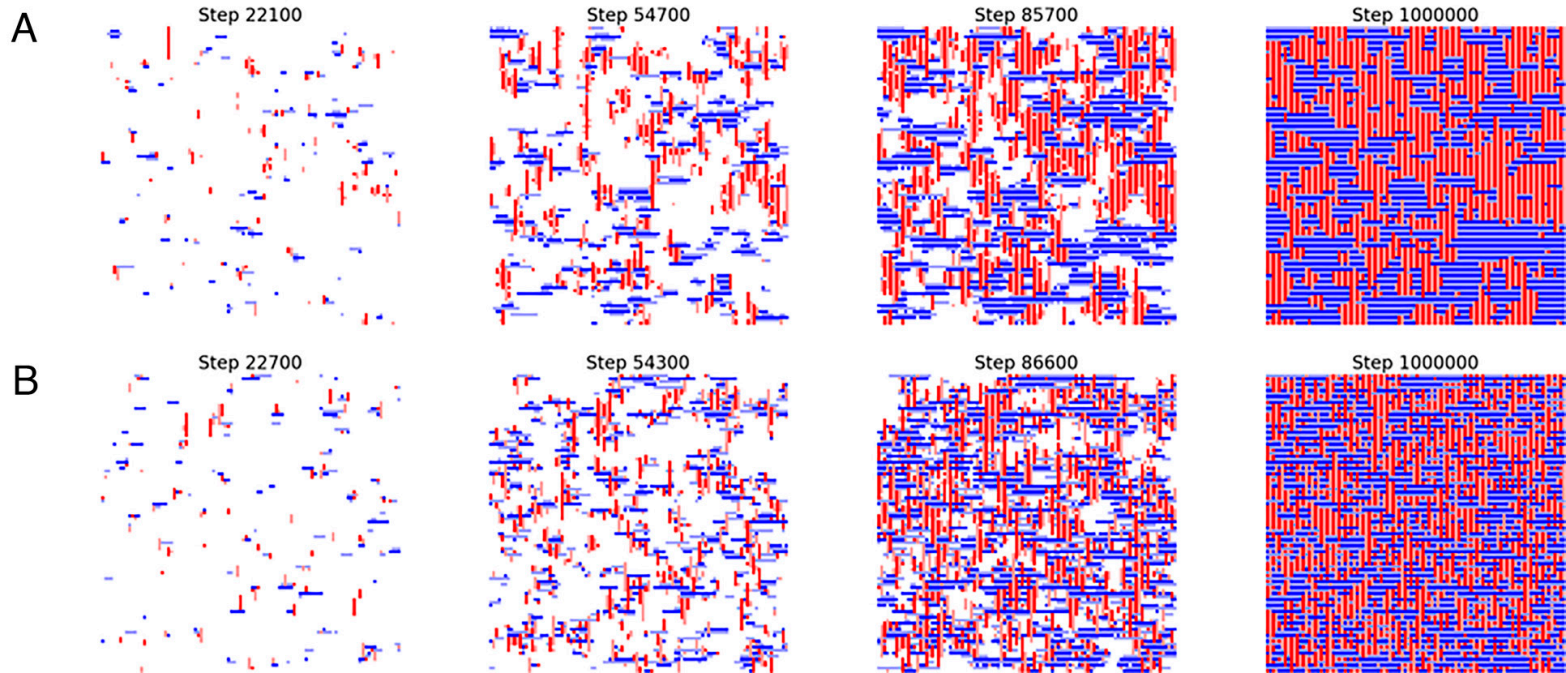
Time-lapse snapshots from Monte Carlo simulations illustrating domain formation at different ε_lateral_ values: (*A*) ε_lateral_ = −0.5 (lateral rodlet interactions included), (*B*) ε_lateral_ = 0.0 (lateral interactions not defined).

Monte Carlo simulations were performed on a two-dimensional lattice to reproduce the observed rodlet bundling and the creation of domain structures (Fig. 5). In our simulations, rodlets appeared to form domain structures when lateral interactions between them were taken into account (Fig. 5*A* and Movie S4). When lateral interactions were not defined, rodlet assemblies appeared to be dispersed (Fig. 5*B* and Movie S5). In these simulations, elongation at the tip was more likely to occur in a bundled rodlet than in a single rodlet (*SI Appendix*, Fig. S3 *A* and *B*). To quantify rodlet orientation, we calculated the angle pair correlation function. Strong lateral interactions yielded larger, more ordered domains, whereas weakening of these interactions led to smaller domains with reduced alignment (*SI Appendix*, Fig. S3*C*). In the presence of strong lateral interactions, counting of the number of events of surface-catalyzed elongation and independent elongation of single rodlets revealed that the former occurred more frequently than the latter (*SI Appendix*, Fig. S3*D*)

## Discussion

We performed bulk analysis and single-rodlet observations by using HS-AFM to elucidate the dynamics of rodlet formation. Examination of the CD spectra revealed that, similar to other class I hydrophobins (21, 27), RolA undergoes a conformational change during the self-assembly of monomers into rodlets composed of β-sheet structures (*SI Appendix*, Fig. S1 *C* and *D*). This result is consistent with that of Pham et al. (14) for the class I hydrophobin MPG1; they found that the rapid progression of nucleation prevented the detection of a lag phase. The shape of the reaction curve for RolA (*SI Appendix*, Fig. S1*C*) is also likely due to a rapid nucleation step. No increase in ThT fluorescence was observed in the absence of an air–water interface (*SI Appendix*, Fig. S1 *E* and *F*), indicating that RolA needed a hydrophobic surface for nucleation. This is consistent with previous studies on RolA and other class I hydrophobins (14, 17, 18). Although the ThT assay allows a kinetic model analysis of the reaction curves, it only provides the apparent reaction rate of the entire system, limiting the scope of the analysis and preventing a detailed investigation of the specific structural changes of RolA and the dynamics of rodlet formation.

By using HS-AFM, we monitored rodlet formation at the single-fibril level in real time and analyzed its conformational dynamics and kinetics. Our observations revealed that RolA spontaneously assembles to form spherical structures on the substrate, which transition into rodlets that elongate further (Fig. 1 *A* and *C*). These structures may represent either RolA monomers in a specific transient conformational state or aggregation-competent oligomers. Although hydrophobins, including RolA, are approximately 10-kDa molecules with diameters of around 3 nm (14, 16), most of their structures consist of flexible loop regions. As a result, the height of the monomers measured by AFM may vary depending on their conformational state. For comparison, in α-synuclein—a 14-kDa intrinsically disordered protein—the height of the monomers measured by AFM ranges from 1 to 5 nm, depending on the structural state (28). Given the size of the observed RolA spherical structures (2.3 nm) (Fig. 1 *C* and *D*), they may thus be either monomers in a particular conformation or small oligomers, but, in either case, they can likely be regarded as precursors of rodlets. HS-AFM also detected spherical structures that did not transition into rodlets (Fig. 1*A*). Because these structures tended to be larger than the rodlet precursors, they might be off-pathway misassembled RolA oligomers or monomers trapped in an incorrect conformational state. HS-AFM analysis also revealed that rodlet ends alternated between growth and pause states (Fig. 1 *F* and *G*). This suggests that the rodlet ends adopt two distinct conformational states: one that allows monomer binding and rodlet elongation and another one that prevents monomer binding, as suggested previously (29, 30). This stepwise mechanism is likely to be universal for amyloid fibril elongation, because it has also been observed in functional amyloids involved in bacterial biofilm development and in pathogenic amyloid formation (29, 31).

Our HS-AFM observations also revealed that rodlets have low polarity in the direction of extension (Fig. 2 *A* and *B*). In general, amyloid fibrils have strong polarity in their direction of extension. For example, HS-AFM and fluorescence microscopy studies of fibrils of well-characterized amyloids such as Aβ, α-synuclein, and Sup35 have shown that they elongate unidirectionally from one end or that elongation rates differ significantly between the two ends (29, 32–37). Highly polarized elongation has also been observed by HS-AFM for the secreted functional amyloid CsgA, which is involved in bacterial biofilm formation (31). Our HS-AFM analysis revealed that RolA rodlets had some degree of polarity but elongated from both ends (Fig. 1 *F* and *G* and 2 *A* and *B*). Bidirectional elongation may provide an advantage in rodlet film formation by allowing a more efficient increase in rodlet mass from a limited number of nuclei, particularly in environments with low nucleation rates.

**Fig. 6.**
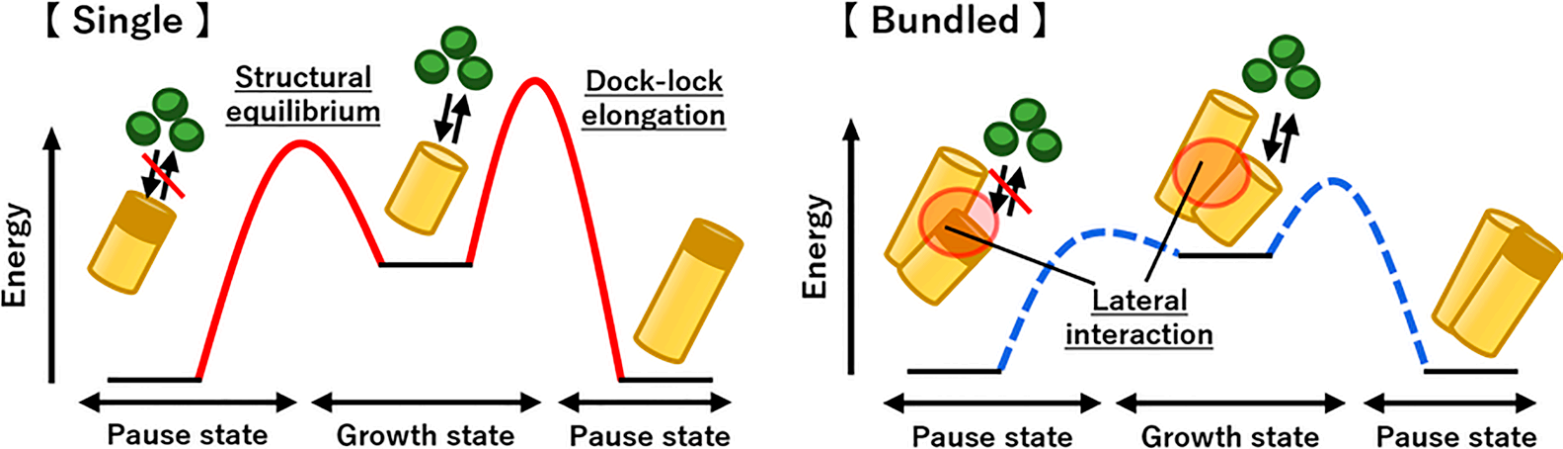
Schematic model of rodlet elongation. Rodlet ends can be either in a growth state (monomers can bind and rodlets can elongate) or in a pause state (monomers cannot bind). Both states are in equilibrium. When rodlets are bundled via lateral interactions between their sides, the energy barrier between the states decreases. Rodlets elongate in a dock–lock manner, and the energy barrier of this reaction also decreases when rodlets are bundled. These decreases contribute to the increase in the elongation rate of bundled rodlets.

One of the most notable findings of this study is that the elongation rate of bundled rodlets was approximately twice that of individual rodlets (Fig. 4*A*), suggesting that interactions between adjacent rodlets promote elongation. This increase in elongation rate is likely due to changes caused in the local environment at the rodlet ends by bundling. Mean dwell and step times were shorter and mean step size and step rate were greater in bundled rodlets than in single rodlets (Fig. 4 *B*–*D* and *SI Appendix*, Table S3). The equilibrium constant was estimated as K_d_ = k_open_/k_close_, where k_open_ = 1/τ_dwell time_, k_close_ = 1/τ_step time_; the K_d_ was 2.12 for bundled rodlets and 2.15 for single rodlets, indicating no significant difference between the two conditions. This suggests that the difference in chemical potential between the growth and pause states remains approximately constant regardless of whether the rodlets are single or bundled. However, the significantly higher frequency of end switching between the pause and growth states in bundled rodlets (Fig. 4 *A*–*C*) suggests a lower energy barrier for this reaction than that in single rodlets (Fig. 6). These results are consistent with the basic concept of catalysis, where the reaction rate increases without changes in the chemical potential of the reactants and products, so the results suggest that rodlets act as catalysts for the structural change of adjacent rodlet ends (Fig. 6). However, this alone does not explain the higher elongation rate in bundled than in single rodlets. Fitting of the data from the ThT assay indicated that RolA elongates in a dock–lock-like manner and that the locking process is the rate-limiting step in the whole reaction (*SI Appendix*, Fig. S1*D*), so rodlet elongation has to be considered as a multistep reaction: [Pause state] ↔ [Growth state] + [Monomer] ↔ [Growth state + Intermediate] → [Elongated rodlet]. In this scenario, the locking process ([Growth state + Intermediate] → [Elongated rodlet]) is also considered to be promoted when rodlets are bundled. The increase in step rate in bundled rodlets (*SI Appendix*, Table S3) suggests a decrease in the energy barrier for the locking process. The rodlet surface can be considered to act as a reaction field that catalyzes both the structural equilibrium of rodlet ends (Growth ↔ Pause) and the locking process, increasing the elongation rate in bundled rodlets (Fig. 4*A*). Three explanations are possible for the molecular mechanism. i) A structural change at the rodlet ends promotes both structural equilibrium and the locking process. Molecular dynamics simulations have suggested that structural fluctuations at the ends of amyloid fibrils influence fibril elongation (38). ii) RolA that undergoes docking but is not locked may interact with neighboring rodlets, thereby influencing elongation (39). iii) RolA monomers could be attached to and concentrated on the rodlet surface, increasing the probability of their interaction with the ends of the adjacent rodlet (docking) (40). In contrast, the locking process is independent of monomer concentration and is therefore unlikely to be subject to this effect (14), but the reduction in the pause state time in bundled rodlets may be explained by such a monomer concentration effect. This effect is easier to understand if amyloid fibril elongation is considered as one-dimensional crystal growth. In crystallization, monomers bind to the crystal surface (terrace), diffuse, and are incorporated into the crystal at kink sites. In a model for amyloid fibril growth, monomers bound to the fibril surface diffuse along the lateral surface and are incorporated into the fibril ends (40). In structures consisting of aligned fibrils of different lengths, the lateral surface of long rodlets can be regarded as corresponding to a terrace and the ends of the short rodlets as corresponding to kink sites. Adsorption of monomers to the lateral surface of rodlets results in a higher local concentration than that in the bulk solution (41), thereby facilitating subsequent rodlet elongation. Although the detailed molecular mechanisms remain unclear, rodlets certainly play a catalytic role in both the structural equilibrium of neighboring rodlet ends and elongation (Fig. 6). Therefore, we suggest calling the RolA rodlet formation pathway “surface-catalyzed elongation,” and we consider that this concept is crucial for understanding rodlet bundling.

Surface catalysis in amyloid aggregation involves secondary nucleation (42), in which nucleation on the fibril surface is strongly promoted; the reverse reaction—oligomer dissociation—is also facilitated, allowing the fibril to act as a catalyst (43). Surface-catalyzed elongation differs from secondary nucleation, because it promotes rodlet elongation rather than the formation of oligomers. Secondary nucleation results in a rapid increase in total fibril mass. Surface-catalyzed elongation may have a limited effect on total fibril mass, because the rodlets formed remain tightly packed as bundles and previously active catalytic sites become buried, preventing a net increase in their number. Surface-catalyzed elongation likely regulates fibril alignment, contributing to the formation of ordered dense rodlet films. To support this hypothesis, we performed Monte Carlo simulations to reproduce rodlet bundling and subsequent domain structure formation. In our simulations, rodlets formed domain structures under conditions of strong lateral interactions (Fig. 5*A*). The appearance of our simulated structures was similar to the result of a coarse-grained simulation performed by Zykwinska et al. (44). These results suggest that our simulations successfully reproduced the long-timescale rodlet film formation process that HS-AFM was unable to fully capture. Further detailed analysis of these simulated results quantitatively demonstrated that lateral interactions of rodlets both promote domain enlargement and enhance alignment (*SI Appendix*, Fig. S3*C*), strengthening the hypothesis that the local phenomena observed by HS-AFM can broadly influence the overall rodlet film formation. Lateral interactions between rodlets are likely to be mediated by specific amino acid residues of individual hydrophobin molecules exposed on the rodlet surface. However, the precise three-dimensional structure of the entire rodlet assembly remains elusive. Further structural analysis is required to elucidate the molecular mechanisms underlying surface-catalyzed elongation.

Bundling and ordered alignment of fibrils are commonly observed in some functional amyloids that coat cell surfaces, such as hydrophobins of filamentous fungi and chaplins of **Streptomyces* coelicolor* (5, 6, 16, 27, 45, 46). In our HS-AFM analysis, we investigated the formation not only of individual rodlets but also of rodlet films from the perspective of the assembly of mature rodlets. Such a mechanism of rodlet film formation may contribute to morphogenesis in particular species by supporting the development of cell-surface architecture.

We used HS-AFM to analyze the rodlet formation dynamics of hydrophobin RolA at the single-fibril level, revealing several intriguing phenomena: i) rodlet ends adopt two distinct structural states: a growth state and a pause state; ii) rodlets elongate bidirectionally while maintaining polarity, although the growth rate difference between the two ends is moderate; and, the most important finding of this study, namely iii) that a previously unrecognized pathway, “surface-catalyzed elongation,” governs structural equilibrium at the rodlet ends and elongation. Because rodlets interact laterally, we propose that surface-catalyzed elongation enhances the efficiency of their assembly. This work represents a conceptual advance in understanding the mechanism of formation of cell-surface architecture by rodlets.

## Materials and Methods

### Purification of RolA.

A strain of *A. oryzae* overexpressing wild-type RolA was created in our previous study (13) and was grown in YPM liquid medium (1% yeast extract, 2% polypeptone, and 2% maltose). In this strain, the *rolA* gene is under the control of the P-*enoA*142 promoter (47), which is strongly induced by maltose. Wild-type RolA was purified according to Terauchi et al. (17) as follows. Conidia were inoculated into YPM liquid medium at 1 × 10^6^ conidia/mL and cultured at 30 °C for 48 h with shaking (160 rpm). The culture broth was filtered through Miracloth (Merck KGaA, Darmstadt, Germany) to separate the mycelia from the culture supernatant. The supernatant was adjusted to pH 8.5 by using 0.1 M Tris solution (pH 10.5), Milli-Q water was added to adjust the electrical conductivity to 1.0 mS/cm, and the solution was applied to a Cellufine Q-500 column (Seikagaku Co., Tokyo, Japan) equilibrated with 5 mM Tris-HCl buffer (pH 9.0). RolA was eluted with a 0 to 0.3 M linear gradient of NaCl. Each fraction was checked by sodium dodecyl sulfate–polyacrylamide gel electrophoresis (SDS-PAGE; 3% stacking gel and 17.5% running gel). The fraction containing RolA (13.6-kDa) was dialyzed against 10 mM sodium citrate buffer (pH 4.0) and applied to an SP-Sepharose Fast Flow column (GE Healthcare, Chicago, IL) equilibrated with the same sodium citrate buffer. RolA was eluted with a 0.05 to 0.3 M linear gradient of NaCl and the fractions (3 mL each) were collected into test tubes containing 1 mL of 100 mM Tris to neutralize. Each fraction was checked by SDS-PAGE, and the fraction containing RolA was dialyzed against 10 mM ammonium acetate (pH 7.0) and lyophilized. All the purification procedures were performed at 4 °C. Before use, lyophilized RolA was dissolved in 10 mM sodium cetate buffer (pH 5.0) and centrifuged for 15 min at 16,000 × g, 4 °C. Protein concentration in the supernatant was determined with a Pierce BCA Protein Assay Kit (Thermo Scientific, Rockford, IL).

### Measurements of CD Spectra.

RolA monomer solution (50 µg/mL) was prepared in 10 mM sodium acetate buffer (pH 5.0). A rodlet suspension was prepared by vortexing the monomer solution for 1 h at room temperature (23 °C). Each suspension (200 µL) was dispensed into a 1-mm quartz cuvette (GL Sciences, Tokyo, Japan). The spectra were measured in a J-725 CD spectrometer (JASCO International, Tokyo, Japan) over a wavelength range of 190 to 250 nm at 23 °C with a bandwidth of 1 nm and step intervals of 1 nm. The sample compartment was continuously flushed with N_2_ gas. The spectra were averaged over 5 scans. The baseline was recorded by using 10 mM sodium acetate buffer (pH 5.0).

### Thioflavin T Assay.

Because ThT specifically binds to rodlets and emits strong fluorescence (14, 18), it allows evaluation of the rodlet formation process. Lyophilized RolA was dissolved in 10 mM sodium acetate buffer (pH 5.0); ThT dissolved in sodium-acetate buffer, along with sodium acetate buffer, and RolA solution, was added to the wells of a black 96-well microplate (Nunclon Delta Surface; Thermo Electron Corporation, Waltham, MA) in that order to a final ThT concentration of 20 µM, and the fluorescence was measured (430 nm excitation/480 nm emission) in a microplate reader (Fluoroskan Ascent, Thermo) with shaking at 600 rpm (shaking diameter = 3 mm) at 30 °C. The model in Eq. **1** below was used to interpret the obtained curves (14):

[1] $$\frac{dM\left(t\right)}{\mathrm{dt}}=\frac{2k_{+}P_{0}m\left(t\right)}{1+\frac{m\left(t\right)}{K_{M}}},$$

where *M*(*t*) is the total rodlet mass, *m*(*t*) is the monomer mass, *P*_0_ is a constant (number of open ends), *k*_+_ is the elongation rate constant, and *K_M_* is the Michaelis constant of elongation, which gives the monomer concentration at which the effect of saturation becomes important. This model, proposed by Pham et al. (14), assumes that elongation proceeds in two steps, with the monomer first “docking” at the rodlet end, followed by structural reorganization (“locking”) to form a stable structure, suggesting that monomer concentration-independent locking becomes rate-limiting when the monomer concentration is sufficiently high. *P* behaves as a constant rather than a time-dependent variable, likely because hydrophobin nucleation occurs specifically at the air–water interface, where the interfacial area constrains the value of *P* and removes its time dependence.

The above ThT assay was used to assess the effect of the air–water interface on rodlet formation. The presence or absence of the interface was controlled by varying the sample volume in each well according to Pham et al. (14). Wells completely filled with sample solution and sealed represented the no air–water interface condition. After 60 min of measurement, half of the solution in each well was removed and the wells were resealed, thus introducing an air–water interface in the wells. ThT fluorescence was then recorded for an additional 60 min.

For the seeding assay, seeds were prepared by vortexing the RolA monomer solution for 60 min. The assay was performed by adding 10% (v/v) of the seed to the sample solution. The wells were completely filled with the sample solution to remove the air–water interface, and fluorescence was recorded for 360 min at 30 °C with shaking at 600 rpm.

### Preparation of Hydrophobically Modified SiO_2_ Substrate.

The silicon substrate surface was hydrophobically modified according to Terauchi et al. (17). First, silicon wafers (p-Si wafers, ≤0.02 Ω; Mitsubishi Material Trading Co. Ltd., Tokyo, Japan) were consecutively ultrasonically cleaned for 15 min each with chloroform, acetone, and 2-propanol to remove microscopic contaminants from the surface. Then, the wafers were cleaned with an ultraviolet ozone cleaner (SKB401Y-01; Sunenergy Co. Ltd., Kanagawa, Japan) for 30 min each on the back and surface to remove organic contamination on the surface, immersed in 0.1% *n*-octyltrichlorosilane solution in chloroform (Tokyo Chemical Industry Co., Ltd., Tokyo, Japan) and left overnight at room temperature (23 °C) to modify the surface. The surface was then dried with N_2_ gas, and the wafers were stored in a desiccator.

### HS-AFM Imaging.

A custom-built HS-AFM instrument (20, 48) equipped with a small cantilever (BL-AC10DS-A2; Olympus, Tokyo, Japan) was operated in tapping mode with the cantilever oscillating at its resonance frequency in liquid (~0.4 MHz) and an amplitude of ~2.0 nm (free oscillation amplitude ~2.5 nm) at room temperature (23 °C). Probes were prepared on top of the cantilevers by electron beam deposition in a field emission scanning electron microscope. HS-AFM scanned 2 × 2 µm of the observation area with 500 × 250 pixels at 12.75 s per frame. Samples were prepared as follows. Rodlet formation on the surface of a droplet was induced by incubating 20 µL RolA solution (3.68 µM) in 10 mM sodium acetate (pH 5.0) on Parafilm for 5 min at room temperature (23 °C). The droplet surface was then pressed against a hydrophobically treated SiO_2_ substrate for 30 s, causing the rodlet to be transferred to the substrate. The substrate with the attached rodlets was placed in the sample chamber of the HS-AFM instrument filled with 10 mM sodium acetate (pH 5.0). The size and shape of the rodlets were determined by HS-AFM and the buffer in the chamber was replaced with 7.35 µM RolA solution (100 μg/mL) in the same buffer. The RolA concentration was set to a sufficient high level based on the results of the ThT assay (*SI Appendix*, Fig. S1*D*) (18), which indicated that at 100 μg/mL RolA its concentration is not a rate-limiting factor for rodlet elongation, whereas the locking process is rate-limiting independent of monomer concentration. Then, rodlet elongation was imaged by HS-AFM for up to 30 min. HS-AFM movies (image sequences) were processed by using ImageJ 1.53e software (https://imagej.nih.gov/ij/) according to Watanabe-Nakayama and Ono (49). From the HS-AFM videos that captured rodlet elongation, the coordinates of all individual rodlet ends were measured by using ImageJ to obtain the time history of their positions. Rodlets were placed into the following categories for analysis: i) those that were already attached to the substrate at the start of the observation period (preexisting); ii) those that were newly formed from monomeric RolA in the chamber and became attached to the stage during the observation period (de novo); iii) those that were alone on the stage (single); and iv) those located next to each other (bundled). In cases where elongation stopped owing to a rodlet collision, we analyzed only the data recorded before the collision. The numbers of rodlets used for measurements are presented in *SI Appendix*, Tables S1–S3. From these time courses, dwell time, step time, and step size were determined and statistically analyzed. All three parameters had typical exponential distributions (Figs. 2 *C*–*E* and 4 *B*–*D* and *SI Appendix*, Fig. S2 *B*–*D*), as expressed by Eq. **2**:

[2] $$N=A\cdot e^{-t/\tau },$$

where *N* is the number of events, *A* is a constant, *t* is the dwell time, step time, or steep size, and *τ* is the mean time or size.

### Monte Carlo Simulations.

Monte Carlo simulations on a two-dimensional lattice were performed to model the formation of the rodlet assemblies and were based on a previous study (44) with modifications. Particles representing short rodlets were placed in the cells and allowed to interact with rodlets present in the neighboring cells. Each cell could take one of three states: empty (0) or containing a vertical or a horizontal rodlet (−1, 1). Monomeric particles were not considered. The simulation started with all lattices empty, meaning that no rodlets were present in the initial state. At each simulation step, one of the empty cells (*i*, *j*) in the lattice was randomly selected and a rodlet was placed into this cell (corresponding to nucleation). If the four neighboring cells were empty, the orientation of the newly placed rodlet was selected randomly (−1 or 1 with probability 1/2 each). The frequency of this random nucleation is governed by the probability *P*. Of several *P* values tested, *P* = 0.005 reproduced the domain structure well (*SI Appendix*, Fig. S3*E*), and therefore we adopted this value. If some rodlets are present in the neighboring cells, the orientation of the newly placed rodlet is determined by their interaction energy (defined below) with the neighboring rodlets. The vector of the newly placed rodlet (*i*, *j*) is *v* and the vector of its neighboring rodlet (*i’*, *j’*) is *w*. The joint vector between the newly placed rodlet and its neighbor is *b* = (*j*−*j’*, *i*−*i’*). When the orientation of the newly placed rodlet aligned with that of the neighboring rodlet (*v* = *w*) and it was placed in the extension direction (*v* is parallel to *b*), the rodlets were considered to have elongated, and the depth of the potential energy between them was defined as ε_elongation_. If *v* = *w* but the new rodlet was placed laterally (*v* is orthogonal to *b*), the depth of the potential energy between them was defined as ε_lateral_. If the orientation of the new rodlet did not align with that of the existing rodlet (*v ≠ w*), the depth of the potential energy was set to 0. Surface-catalyzed elongation gave ε_elongation_ + ε_lateral_. The frequency of nucleation when neighboring rodlets were present was set to 0.1 if the newly placed rodlet was aligned with the extension direction, and 0.005 otherwise. Simulations were performed with the following parameters: lattice size, 100 × 100; maximum number of simulation steps, 1,000,000 (the simulation ended when all cells were filled with rodlets); and temperature, *k*_B_*T* = 0.1, where *k*_B_ is the Boltzmann constant. Six simulations were performed with ε_elongation_ fixed at −1.0 and ε_lateral_ varied (0.0, −0.01, −0.1, −0.2, −0.5, −1.0). The time evolution of the Monte Carlo simulations was governed by the Metropolis algorithm (50). To evaluate the degree of rodlet orientation, we calculated the angle pair correlation function (51, 52). The size and degree of alignment of a domain structure can be evaluated by examining how well the direction of rods correlates with that of distant rods. The angle pair correlation function is defined in Eq. **3**:

[3] $$G_{2}\left(r\right)={\langle \text{cos}2\left(\theta_{i}-\theta_{j}\right)\rangle }_{\left|r_{i}-r_{j}\right|},$$

where *θ_i_* and *θ_j_* are the angles of rods *i* and *j*, respectively, and |*r*_i_ −*r*_j_| is the Euclidean distance between them. *G*_2_(*r*) = 1 indicates an ordered structure in which all rods are perfectly oriented, whereas *G*_2_(*r*) = 0 indicates a disordered structure with randomly oriented rods.

To estimate the elongation increment per step, a simplified system was designed with 10 rodlets (length = 100) placed at equal intervals and additional short rodlets (length = 2) positioned laterally in contact. The same constraints for rodlet elongation as above were applied; nucleation was turned off to isolate the effect of elongation.

## Supplementary Material

Appendix 01 (PDF)

Movie S1. HS-AFM video of the entire field of view. HS-AFM scanned 2 × 2 µm of the observation area at 12.75 s per frame. The video is played back at ×50 higher speed.

Movie S2. HS-AFM video of rodlet elongation from both ends. Enlarged 2 × 2 µm scan video at 12.75 s per frame. The video is played back at ×50 higher speed.

Movie S3. HS-AFM video of rodlet bundling. Enlarged 2 × 2 µm scan video at 12.75 s per frame. The video is played back at ×50 higher speed.

Movie S4. Time-lapse video of Monte Carlo simulation considering lateral interactions between rodlets. ε_elongation_, −1.0; ε_lateral_, −0.5; lattice size, 100 × 100; number of simulation steps, 1,000,000; *k*_B_*T* = 0.1.

Movie S5. Time-lapse video of Monte Carlo simulation assuming no lateral interactions between rodlets. ε_elongation_, −1.0; ε_lateral_, 0.0; lattice size, 100 × 100; number of simulation steps, 1,000,000; *k*_B_*T* = 0.1.

## Data Availability

Study data are included in the article and/or supporting information.
