# Supplementary material for: High-speed atomic force microscopy reveals a surface-catalyzed elongation mechanism of the fungal functional amyloid hydrophobin RolA
Source: Proc Natl Acad Sci U S A. 2026 Feb 12;123(7):e2523502123. doi: 10.1073/pnas.2523502123 (PMC12912973; doi:10.1073/pnas.2523502123)
Supplement: Supplementary file 1 — Appendix 01 (PDF) [file pnas.2523502123.sapp.pdf]

Supporting Information for

High-speed atomic force microscopy reveals surface-catalyzed elongation mechanism of the fungal functional amyloid, hydrophobin RolA

Nao Takahashi, Tatsuya Kimura, Yuki Terauchi, Takumi Tanaka, Natsuki Abe, Akira Yoshimi, Takahiro Watanabe-Nakayama, Keietsu Abe

Keietsu Abe

Email: keietsu.abe.b5@tohoku.ac.jp

This PDF file includes:

Figures S1 to S3

Tables S1 to S3

Legends for Movies S1 to S5

Other supporting materials for this manuscript include the following:

Movies S1 to S5

Fig. S1.

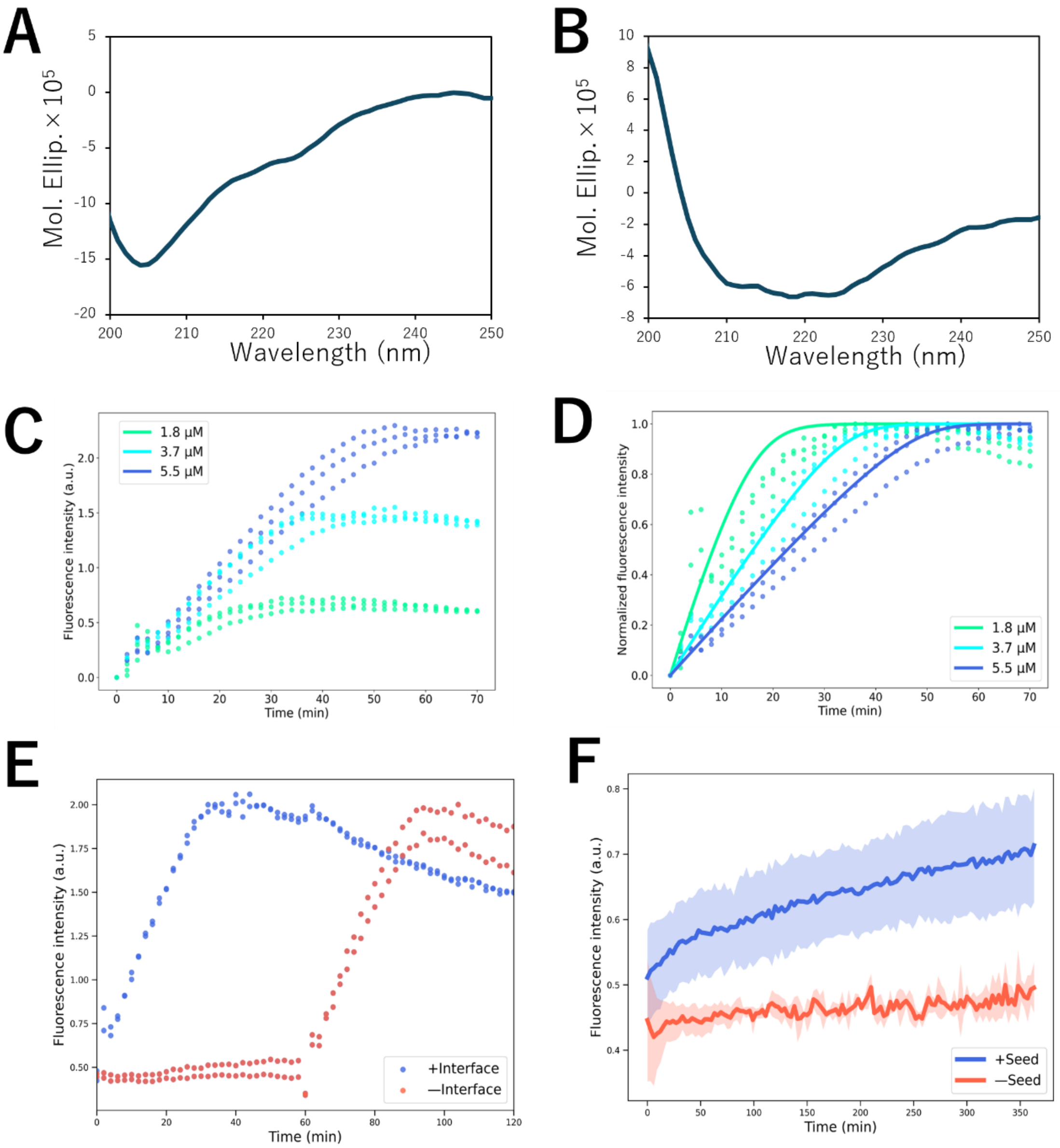

Formation of RolA rodlets in bulk solution. (A, B) CD spectra of 50 μg/ml RolA in the monomeric state (A) and rodlet state (B), indicating a conformational change through self-assembly. (C, D) Time course of ThT fluorescence at different initial RolA concentrations at 30 °C with shaking: raw data (C) and normalized data (D). Dotted lines, measured data; solid lines, results of fitting using Eq. 1 (see Materials and Methods). (E) ThT assay in the presence (blue dots) or the initial absence (red dots) of an air–water interface, which was introduced in the latter case at 60 min. (F) Seeding assay in the absence of an air–water interface (mean ± S.D.).

Fig. S2.

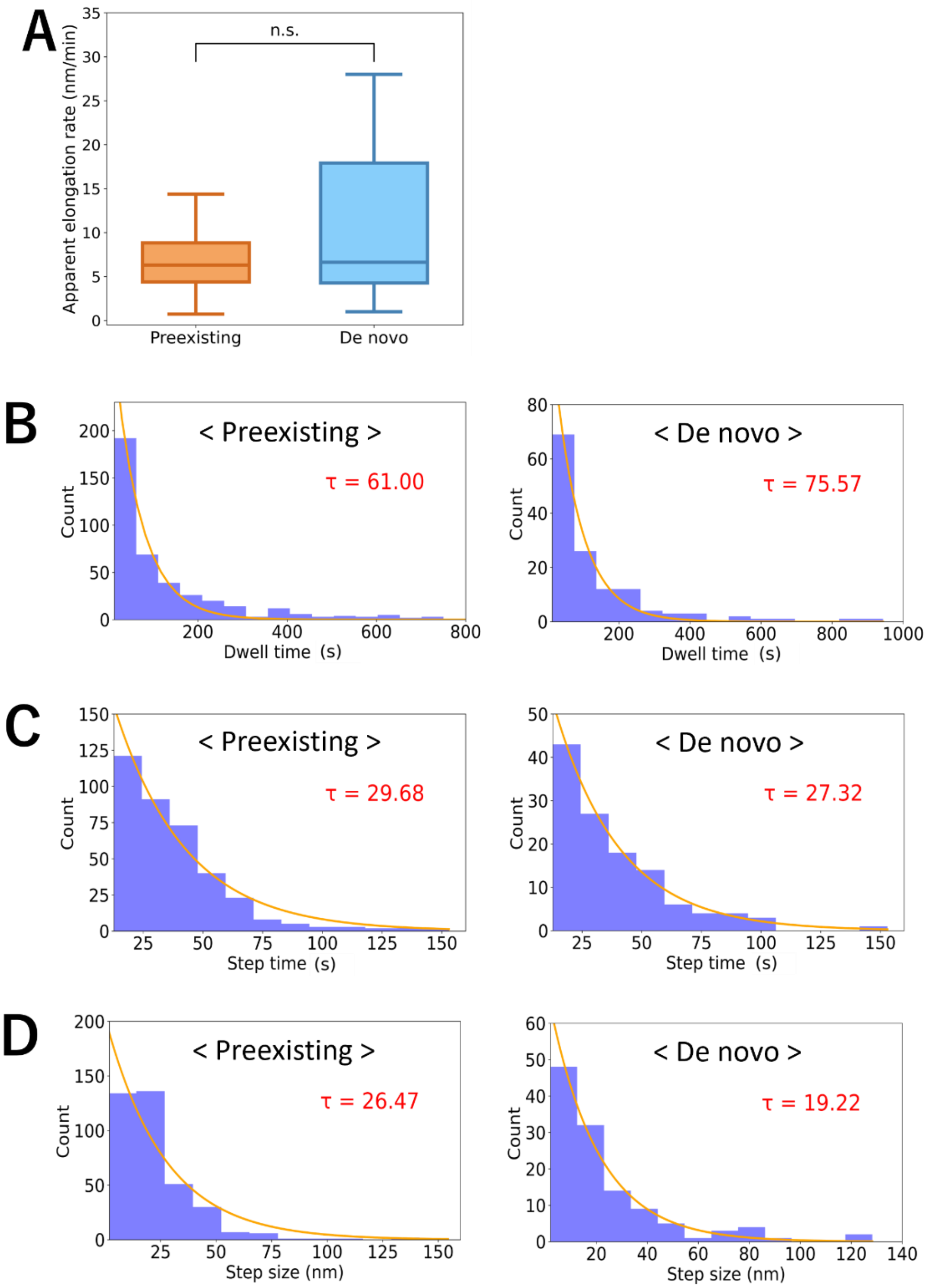

Elongation kinetics of preexisting and de novo rodlets. (A) Apparent elongation rates. Boxes extend from the 25th to 75th percentiles. The line in each box indicates the median. Whiskers reach out to the most distant point that's still within 1.5 times the interquartile range. Statistical significance was evaluated by using the Brunner–Munzel test; n.s., not significant. (B–D) Distribution of dwell time (B), step time (C), and single-step size (D), with exponential fits (lines) giving mean values of τ shown in each panel and Table S1.

Fig. S3.

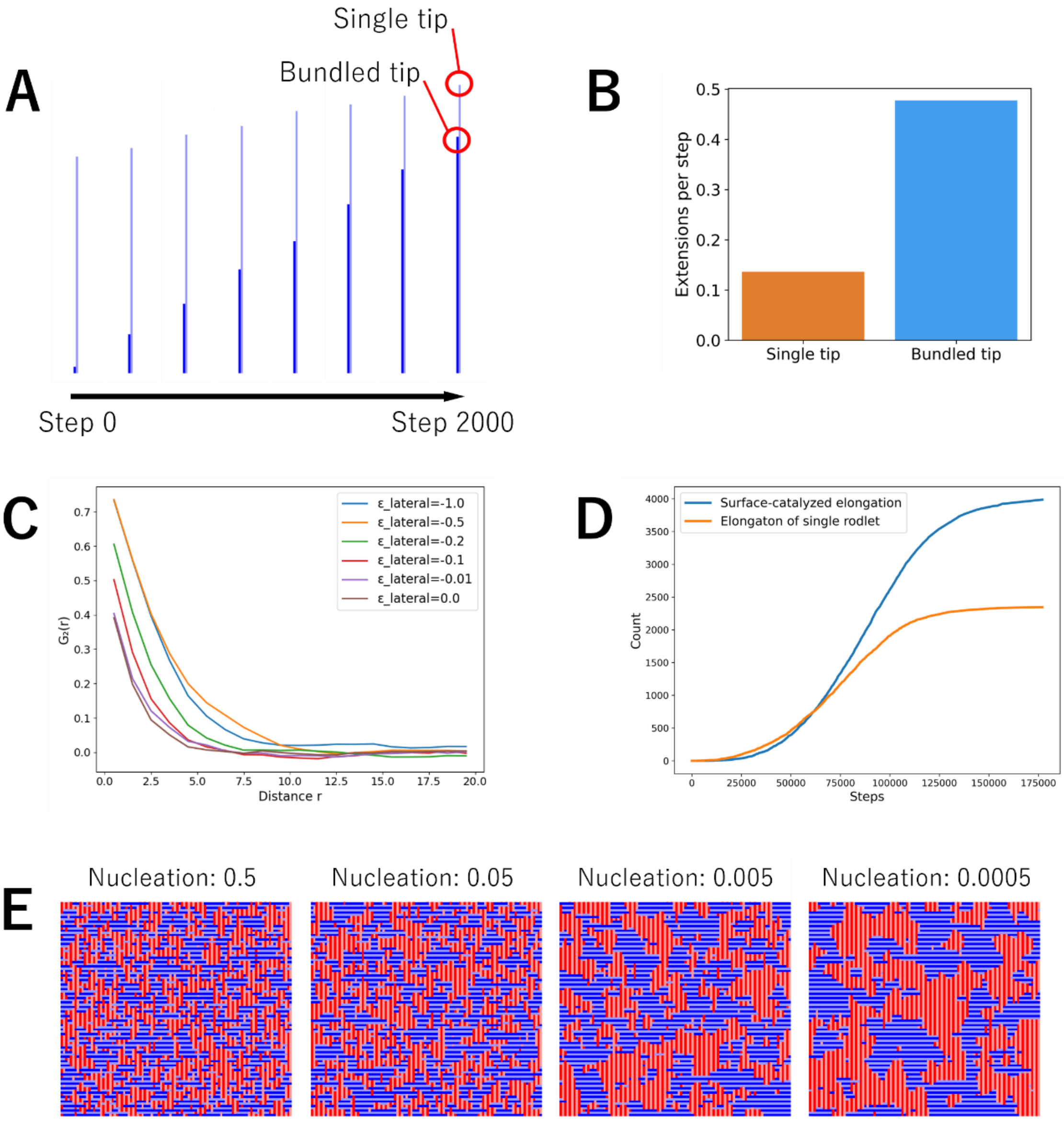

Effects of lateral rodlet interactions on elongation and rodlet alignment in the Monte Carlo simulations. (A) Representative time-lapse image of elongation of both single and bundled tips. (B) Extensions per simulation step. (C) Angle pair correlation function $G_2(r)$ defined in Eq.3 (see Materials and Methods) at different ε_lateral values. (D) Contribution of surface-catalyzed elongation to domain formation. (E) Time-lapse snapshots from Monte Carlo simulations at different nucleation frequencies.

Table S1.

Kinetic parameters of preexisting and de novo rodlets

	Preexisting	De novo	*P* value*
Number of rodlets analyzed	51	24	–
Total number of steps analyzed	411	137	–
Apparent elongation rate (nm/min)	7.9	11.6	0.609
Mean dwell time (s)	61.0	75.6	0.747
Mean step time (s)	29.7	27.3	0.872
Mean step size (nm)	26.5	19.2	0.250
Step rate (nm/min)	9.0	8.8	0.462

* *P* values were calculated by using the Brunner–Munzel test.

Table S2.

Kinetic parameters of fast and slow ends

	Fast	Slow	*P* value*
Number of rodlets analyzed	20	20	–
Total number of steps analyzed	158	126	–
Apparent elongation rate (nm/min)	12.6	5.2	8.33×10^{-5}
Mean dwell time (s)	57.6	95.7	0.0556
Mean step time (s)	27.6	25.9	0.432
Mean step size (nm)	21.7	18.8	0.0682
Step rate (nm/min)	9.2	8.5	0.0906

* *P* values were calculated by using the Brunner–Munzel test.

Table S3.

Kinetic parameters of bundled and single rodlets

	Bundled	Single	*P* value*
Number of rodlets analyzed	13	62	–
Total number of steps analyzed	105	441	–
Apparent elongation rate (nm/min)	13.9	7.4	0.0140
Mean dwell time (s)	43.6	67.1	0.0244
Mean step time (s)	20.6	31.2	0.0131
Mean step size (nm)	36.4	22.2	1.53×10^{-4}
Step rate (nm/min)	17.9	10.4	1.31×10^{-7}

* *P* values were calculated by using the Brunner–Munzel test.

Movie S1 (separate file).

HS-AFM video of the entire field of view. HS-AFM scanned 2 × 2 μm of the observation area at 12.75 s per frame. The video is played back at ×50 higher speed.

Movie S2 (separate file).

HS-AFM video of rodlet elongation from both ends. Enlarged 2 × 2 μm scan video at 12.75 s per frame. The video is played back at ×50 higher speed.

Movie S3 (separate file).

HS-AFM video of rodlet bundling. Enlarged 2 × 2 μm scan video at 12.75 s per frame. The video is played back at ×50 higher speed.

Movie S4 (separate file).

Time-lapse video of Monte Carlo simulation considering lateral interactions between rodlets. $\varepsilon_{elongation}$, −1.0; $\varepsilon_{lateral}$, −0.5; lattice size, 100 × 100; number of simulation steps, 1,000,000; $k_B T$ = 0.1.

Movie S5 (separate file).

Time-lapse video of Monte Carlo simulation assuming no lateral interactions between rodlets. $\varepsilon_{elongation}$, −1.0; $\varepsilon_{lateral}$, 0.0; lattice size, 100 × 100; number of simulation steps, 1,000,000; $k_B T$ = 0.1.
